# Use of mesenchymal stem cells seeded on the scaffold in articular cartilage repair

**DOI:** 10.1186/s41232-018-0061-1

**Published:** 2018-03-12

**Authors:** Kaoru Yamagata, Shingo Nakayamada, Yoshiya Tanaka

**Affiliations:** The First Department of Internal Medicine, School of Medicine, University of Occupational and Environmental Health, Japan, 1-1 Iseigaoka, Yahata-nishi-ku, Kitakyushu, 807-8555 Japan

**Keywords:** Articular cartilage, Mesenchymal stem cells (MSCs), Scaffold, Poly-lactic-co-glycolic acids (PLGA), Hyaluronic acid (HA)

## Abstract

Articular cartilage has poor capacity for repair. Once damaged, they degenerate, causing functional impairment of joints. Allogeneic cartilage transplantation has been performed for functional recovery of articular cartilage. However, there is only a limited amount of articular cartilage available for transplantation. Mesenchymal stem cells (MSCs) could be potentially suitable for local implantation. MSCs can differentiate into chondrocytes. Several studies have demonstrated the therapeutic potential of MSCs in the repair of articular cartilage in animal models of articular cartilage damage and in patients with damaged articular cartilage. To boost post-implantation MSC differentiation into chondrocytes, the alternative delivery methods by scaffolds, using hyaluronic acid (HA) or poly-lactic-co-glycolic-acid (PLGA), have developed. In this review, we report recent data on the repair of articular cartilage and discuss future developments.

## Background

The articular cartilage plays an important role in the smooth motion of joints. Aging is associated with thinning of the articular cartilage tissue and reduction of its function. Aging is also associated with diminished physical activity, leading to impaired activity of daily living (ADL) and quality of life (QOL). The articular cartilage is a structurally unique tissue, lacking blood vessels and nerves, and is considered to be in a low-nutrient, low-oxygen environment. Furthermore, the inflammatory milieu breaks down the cartilage matrix and induces apoptosis of chondrocytes, leading to irreversible defect in the cartilage, a process that is currently difficult to repair in patients with cartilage degenerative diseases, including rheumatoid arthritis (RA) and osteoarthritis (OA). While certain managements are available to alleviate pain or recover cartilage function, these do not result in recovery once the articular cartilage is damaged. Thus, there is a need to design new techniques for repair of articular cartilage and hence to improve ADL and QOL. In fact, several procedures, such as joint replacement, allogeneic chondrocyte implantation, and implantation of mesenchymal stem cells (MSCs) seeded on scaffold, have been used in regenerative medicine of the articular cartilage.

Joint replacement bears a heavy burden on patients, and some undesirable effects on the surrounding tissues are sometimes unavoidable. Two types of osteochondral transplantations are considered as alternative techniques. One is autologous osteochondral transplantation, which involves grafting articular cartilage taken from healthy subjects into the affected area [1]. The pathological features of the articular cartilage improve over a short term, whereas the long-term effects are inconsistent [1, 2]. The other technique is allogeneic osteochondral transplantation with the goal of repairing widespread defect in the articular cartilage. In fact, this technique provides improvement of the articular cartilage [3]. However, there remain several issues that need to be discussed, such as the need for adaptation of donor’s graft size to the recipient one, assessment of the general health condition, with or without infection [4, 5].

Autologous chondrocyte implantation has been tried also as an alternative strategy. The aim of such treatment is to repair the articular cartilage via implantation of chondrocytes into the affected area after in vitro proliferation of samples prepared from healthy articular cartilage [6]. It has been reported that chondrocytes seeded on a scaffold then implanted into the cartilaginous defect can result in the repair of articular cartilage of the knee and ankle tissues within 7–13 and 2–5 years, respectively [7–9]. However, this method has its limitation especially with the use of less than the recommended number of chondrocytes during the implantation process; such cells lose their ability to produce cartilage extracellular matrix (ECM) like hyaline cartilage due to the dedifferentiation of these cells [10–12].

Another promising strategy that has been tested recently is the use of a scaffold alone or bone marrow-derived MSCs seeded on scaffold. MSCs reside in many types of tissues, including bone marrow, adipose, or synovium, and are easy to isolate from these organs. In vitro studies showed that bone marrow-derived MSCs can differentiate into various mesenchymal lineages, including chondrocytes [13]. In vivo studies showed that MSCs contribute to the coverage of articular cartilage, indicating that MSCs are proper tool for implantation to repair the articular cartilage [14–16]. Recently, different types of MSCs other than bone marrow-derived MSCs, including ones derived from synovial tissue, peripheral blood, periosteum, or adipose tissue, have been focused in terms of articular cartilage repair [17–20]. The accumulating evidences demonstrate potential utility of MSCs in the repair of articular cartilage. In particular, it is easy to take large amounts of adipose-derived MSCs (ASC) from fat tissue. However, ability to differentiation of ASC into chondrocytes is poor [21, 22].

In this review, we introduce recent evidences and current status based on mechanism of chondrocyte differentiation and regeneration of the articular cartilage, and then discuss future prospects.

### Damage of articular cartilage reflects physical disorder in RA

RA is a systemic autoimmune disease characterized by chronic inflammatory synovitis and progressive joint destruction, which is associated with serious morbidity and mortality [23–25]. Without appropriate treatment, the patients suffer heavy physical disorder associated with limited joint function [24, 25]. Especially, destruction of the articular cartilage, but not bone tissue, correlates with the physical disorder of RA [26, 27]. Clinical or structural remission has recently become an achievable goal through the use of methotrexate (MTX) as the first line disease modifying antirheumatic drug, in addition to tumor necrosis factor (TNF) inhibitors, interleukin-6 (IL-6) inhibitors and cytotoxic T-lymphocyte-associated antigen 4 immunoglobulin fusion protein (CTLA-4Ig), or small-molecular compounds that target Janus kinase (JAK) [28–30]. In this regard, rapid and effective induction of remission is a prerequisite for halting the process of joint destruction. However, it is still difficult to repair damaged or degenerated articular cartilage. Therefore, there is a need for novel treatment strategies, such as regenerative medicine.

### Mesenchymal stem cells can differentiate into chondrocytes

The articular cartilage covering the bone heads is composed of chondrocytes and cartilage ECM, which is comprised of aggrecan, proteoglycan, type II, IX, and XI collagen. These tissues, however, show poor self-repair capability. Damage or loss of these tissues often results in functional disorder such as OA. At present, autologous cartilage tissue implantation is applied for functional recovery of articular cartilage tissue [31], but unfortunately, this treatment has the following demerits. First, only a limited amount of osteochondral tissue can be prepared from the patients. Second, the implantation further hurts the residual healthy articular cartilage. Based on the above fact, there is certainly a need to develop novel therapies that can prevent and promote repair of damaged articular cartilage.

Different scaffolds have been designed as the delivery system for the repair of articular cartilage. MSCs reside in various types of tissues, including bone marrow, adipose, synovium, cartilage tissue, and placenta. These cells can differentiate into different types of cells that constitute the joints, including osteoblasts, osteocytes, tenocytes, adipocytes, and chondrocytes [13]. It is anticipated that the use of MSCs residing on scaffolds may help in the regeneration/repair of degenerated or damaged articular cartilage. However, endogenous MSCs have poor ability to repair articular cartilage. Although MSCs are injected intravenously (IV), intra-articularly (IA), or intra-peritoneally (IP), the cells diffuse into the peripheral blood and reside in non-affected area [32–35]. Consequently, such implantation has little effect on the phenotype of the destroyed cartilage tissue. In order to overcome this problem, the transplantation of MSC formed in three dimensional structures, such as cell aggregates and sheets, have been tried [36].

On the other hand, other biological functions of MSCs, such as anti-inflammation, anti-fibrosis, migration, and proliferation, have been reported [32, 33, 37, 38], indicating critical role of MSCs instead of chondrocyte differentiation in cell therapies. In this review, we focus on chondrogenesis related to the repair of articular cartilage.

### Chondrogenic differentiation between the 2D and 3D cultures

MSCs in the living body reside in 3dimensional (3D) circumstance. To make implanted MSCs reside in 3D, pre-implantation (IMP) MSCs should be set at 3D, in this case MSCs are seeded on various types of scaffolds. 3D scaffold should be special material, that mimic circumstance in the living body and is proper for cell adhesion, differentiation, proliferation, and formation of cartilage ECM [39].

After harton’s jelly (WT)-MSCs were cultured with chondrocyte differentiation medium over 21 days, transcriptional activity of *type II collagen* gene was increased in the culture of 2D (PLGA free monolayer) or 3D with PLGA scaffold [39]. Expression of both *type I collagen* (an osteoblast marker) and *type III collagen* (a fibrocartilage marker) were decreased in 3D whereas their expression were increased in 2D. This indicates that MSCs in 3D, but not 2D, may play role in the formation of hyaline cartilage, but not fibrocartilage or bone tissue.

We have reported that MSCs were cultured with cell growth medium in 2D with cell monolayer (PLGA free) or 3D with PLGA plug scaffold [40]. 3D culture at day 7, but not 2D, up-regulated SOX9 (master regulators of bone and cartilage differentiation). MSCs in 3D culture at day 14, but not 2D, showed positive staining for proteoglycan by safranin O staining. Taken together, 3D-based PLGA promotes efficiently the chondrocyte differentiation of MSCs in vitro without any cytokine stimulation.

Other group showed that compared with 2D culture with MSCs monolayer, collagen-based sponge could enhance differentiation of MSCs into chondrocyte in vitro. This indicates that type II collagen as a cartilage ECM contributes to differentiation of MSCs into chondrocytes.

Thus, these results show significance and generality of 3D MSCs culture with scaffold in chondrogenesis.

### PLGA scaffold is required for the repair of articular cartilages

The purpose of implantation is for MSCs to efficiently differentiate into chondrocytes, then express large amounts of cartilage ECM, form hyaline cartilage, and then assimilate into the surrounding tissues. First, a scaffold is required for MSCs to reside on the damaged articular cartilage. Poly-lactic-co-glycolic acids (PLGA) is representative commonly used scaffold composed of both poly-glycolic acid (PGA) and poly-lactic acid (PLA). PLGA has several advantages, such as controlled biodegradability, i.e. it disintegrate in the living body, low immunogenicity, efficient carrier of drugs to the target tissue, forms a scaffold for regeneration of cartilage defect through the support of cell residence and cell differentiation.

Implantation of PLGA alone into the affected joints of a rabbit model of osteochondral defect results in satisfactory repair of the bone and cartilage tissues and results in adequate cover of the defect with cartilage tissue [41]. This finding indicates that endogenous MSCs can adhere to PLGA, and then help in the repair of articular damage. Another in vitro study showed that MSCs seeded on PLGA can differentiate into chondrocytes without any cytokine stimulation [40]. These data emphasize the utility of PLGA as a MSC scaffold to achieve efficient repair of the articular cartilage. On the other hand, bone marrow-derived MSCs obtained from *IL-1Ra* gene knockout mice, which mimic various pathological conditions including RA, have low capacity for self-renewal or differentiation into osteoblasts compared to the wild-type mice [42]. It is possible that MSCs from RA patients also have poor capacity for differentiation. Thus, it is preferable perhaps to co-implant normal and exogenous MSCs, but not endogenous MSCs, with a scaffold into the affected area in order to achieve a better repair of the articular cartilage in RA. Another study reported the finding of positive staining for proteoglycan in the affected region and the formation of hyaline cartilage-like tissue after implantation of MSC sheet-coated PLGA+MSCs into the cartilage defect into the smooth white tissue of rabbits [43].

While the scaffold enhances residence of MSCs into the local tissue, this can be augmented by the addition of cytokines. For instance, PLGA with transforming growth factor-β3 (TGF-β3) enhanced MSC differentiation into chondrocytes, while implantation of PLGA with stromal-derived factor-1α (SDF-1α) into resulted in repair of the articular cartilage [44, 45]. Thus, implantation of PLGA combined with various cytokines enhances more efficient differentiation of MSCs into articular cartilage.

MSC implantation is relatively safe. One study reported lack of any oncogenesis or infection at 5–137 months after MSC implantation [46]. On the other hand, implantation of polyglycolic acid-hyaluronan with MSCs also induced repair of the damaged articular cartilage [43]. To date, however, the use of PLGA for the repair of articular cartilage remains experimental. Thus, more efficient tools are needed in the future.

### Collagen scaffold provides the repair of articular cartilages

Collagen molecules are major components of cartilage ECM, and degraded by collagenases in the living body. Collagen-based material provides proper circumstance for chondrocyte differentiation. Thus, the scaffold is commonly applied for repair strategy of articular cartilage.

Li et al. have reported utility of special tool in the repair of articular cartilage [47]. After rabbit MSCs and collagen are capsuled with microsphere, the tool are applied to implantation into affected area of the osteochondral defect of rabbit. This procedure provided positive staining for type II collagen and glycosaminoglycan (CAG), suggesting formation of hyaline-like tissue. Further, implantation of collagen scaffold alone introduces the repair the osteochondral defect [48]. This finding indicates that the scaffold promotes spontaneous differentiation of endogenous MSCs into chondrocytes.

On the other hand, clinical applications have been tried energetically in addition to studies using animal model. Implantation of collagen gel and MSCs into the athlete, who suffers from knee’s pain, results in the formation of hyaline-like tissue, and functional recovery of the articular cartilage [49]. Collectively, these evidences emphasize that collagen materials are a proper and promising scaffold for the repair of the articular cartilage.

### Gelatin scaffold is required for the repair of articular cartilages

Hydrogel is 3D polymeric material that can retain large amount of water. The scaffold provides good biocompatibility and can have an affinity with growth factor or cells, such as MSCs. To date hydrogel scaffolds, including agarose or gelatin, have been applied to implantation into the articular cartilage defect with the goal of cartilage repair.

Agarose is polysaccharide composed from the residue of L- and D-galactose. Previously agarose-based 3D-cultures have been performed as a scaffold of MSCs to promote in vitro MSCs chondrogenesis [50]. Implantation of agarose and MSCs into the articular cartilage defect of rabbit resulted in positive staining for type II collagen and proteoglycan, providing the repair of articular cartilage [51]. On the other hand, another group reported the agarose implantation may inhibit spontaneous repair of articular cartilage and further accumulate in the living body due to weak biodegradability. Therefore, this strategy might not been proper for in vivo trial related on the repair of cartilage tissue.

Gelatin is synthesized from denatured collagen, exhibits cell-adhesion and has been be applied in a variety of scaffolds. Thus, gelatin is biodegradable and a promising scaffold for regenerative medicine of articular cartilage.

Ponticiello et al. have reported that human MSCs were seeded on gelatin sponge, and cultured for 21 day, showing type II collagen staining [52]. After that, the MSCs were implanted into the osteochondral defect of rabbits. Gelatin and MSCs were observed to be very biocompatible, with no evidence of immune response or lymphocytic infiltration at the site. Gelatin is a promising candidate as a carrier matrix for MSC-based cartilage regeneration.

On the other hand, gelatin has disadvantage, such as weakness to mechanical stress. Chemical modification of gelatin via cross-linking with visible light improved the weakness to the stress [53]. In fact, implantation of MSCs seeded on cross-linking gelatin into the osteochondral defect of rabbits provides the repair for the affected area [54]. Taken together, gelatin is an appropriate material to repair articular cartilage applied with MSCs.

### Other scaffolds that contribute to the repair of articular cartilage

MSC scaffolds other than PLGA, collagen, or gelatin, such as tricalcium (TCP), PLA, hyaluronic acid (HA), PGA, and fibrin glue, have also been used for implantation into the articular cartilage defect in experimental animal models (Table 1). PLGA is composed of PLA and PGA whilst PGA-hyaluronan is predominantly comprised of PGA and hyaluronan. The both material show biodegradability and help in enhanced residence of MSCs at affected areas. PLGA-based TGF-β3-releasing microspheres is used in terms of the following. PLGA is gradually disintegrate in the living body, subsequently result in release of TGF-β3 and efficient cytokine effect over the long-term. As a result, implanted MSCs are subjected to chondrocyte differentiation.

**Table 1:** Application of MSC seeded onto various types of scaffolds into animal models of articular cartilage defect

Design for Implantation	Animal model	Follow-up period (months)	Finding	Ref.
BM-MSC seeded on TCP scaffold	Sheep	6	Proteoglycan and type II collagen	[66]
BM-MSC seeded on PLA scaffold	Dog	1.5	Coverage of chondral defect	[67]
BM-MSC seeded on HA scaffold	Mini-pig	3	Coverage of chondral defect	[14]
BMDC seeded on HA scaffold	Goat	6	Coverage of chondral defect, proteoglycan and type II collagen	[55]
BM-MSC seeded on type I collagen scaffold	Sheep	6	Hyaline-like cartilage	[15]
BMDC seeded on PGA or PLGA scaffold	Sheep	3	Hyaline-like cartilage	[16]
BM-MSC seeded on type I collagen scaffold	Sheep	12	Type II collagen	[68]
BM-MSC seeded on HA scaffold	Horse	12	No difference in chondral surface	[56]
BM-MSC seeded on type II collagen scaffold	Pig	2	Hyaline-like cartilage	[69]
BM-MSC suspended in fibrin glue	Goat	6	Improved cartilage tissue	[70]
BM-MSC seeded on PGA-hyaluronan	Rabbit	1.5	Hyaline-like cartilage	[71]
BM-MSC sheet-encapsulated MSC on PLGA scaffold	Rabbit	3	Hyaline-like cartilage	[43]
MSC seeded on PLGA-based TGF-β3-releasing microspheres	Mice	1.5	Hyaline-like cartilage	[72]

*BM-MSC* bone marrow-derived mesenchymal stem cells, *TCP* tricalcium phosphate, *PLA* polylactic acid, *HA* hyaluronic acid, *PGA* polyglycolic acid, *PLGA* poly-lactic and co-glycolic acids, *TGF-β3* transforming growth factor-β3

HA has been used frequently for implantation of MSCs. Implantation of MSCs-HA into the knee joints of pigs with partial defect in the articular cartilage was followed by efficient covering of the cartilage tissue at 12 weeks followed by the formation of hyaline cartilage-like tissue [14]. However this effect was limited after application of HA alone. Saw et al. [55] reported that the amounts of type II collagen and proteoglycan increased in cartilage defects around the femur tissue after implantation of HA and bone marrow-derived cells (BMDC) in goats. A similar procedure was conducted in pigs. However there was no difference in the repair process of articular cartilage based on MRI imaging between HA and HA + MSC groups at 1 year after implantations [56]. These findings suggest that the efficacy of implantation depends on body size. Further studies to examine changes in cell numbers time of implantation and the implantation tool are required.

Several studies have described the implantation of scaffold and MSCs into affected area in patients with damaged articular cartilage (Table 2). The MRI and arthroscopic findings in patients who had undergone implantation of HA and BMDC with MSCs into the injured joint area showed the formation of new hyaline cartilage-like tissue, which assimilated later into the surrounding tissues within 24 months [57, 58]. Biopsy specimen from these areas showed dense staining for proteoglycan and type II collagen or faint staining for type I collagen, confirming the repair of articular cartilage observed on the MRI images and that the repaired tissue is hyaline cartilage tissue. However, in some cases the results have been the opposite of what was expected. For example, implantation of HA-BMDC-MSCs into the talus was later found to result in the formation of irregular cartilage-like tissue by MRI with little or no assimilation with the residual articular cartilage [59]. Further instrument for implantation is required for the repair of articular cartilage in the affected region.

**Table 2:** Application of MSC seeded onto different types of scaffolds into patients with damaged articular cartilage

Technique	n; Sex; Age (years) (mean ± SD)	Follow-up period (months)	Finding	Ref.
BM-MSC in type I collagen gel	1; M (31)	12	Hyaline-like cartilage	[49]
BM-MSC within type I collagen gel on a collagen scaffold seeded on PLA scaffold	3; 2 M, 1F (32–45)	18	Coverage of chondral defect	[73]
BMDC suspended in collagen or seeded on HA scaffold	48; 27 M, 21F (28 ± 9)	24–35	Coverage of chondral defect and hypertrophic cartilage	[57]
BMDC seeded on HA scaffold supplemented with platelet-rich fibrin	20; 12 M, 8F (28 ± 9)	29 ± 4	Proteoglycan and type II collagen	[58]
BMDC seeded on HA scaffold supplemented with platelet-rich fibrin	81; 47 M, 34F (30 ± 8)	59 ± 26	Hyaline-like cartilage	[74]
BM-MSC within platelet-rich fibrin glue	5; 4 M, 1F (25)	12	Coverage of chondral defect	[75]
BM-MSC covered by periosteum	72; 38 M, 34F (44 ± 11)	24	Aggrecan and type II collagen	[76]
BMDC with batroxobin covered by type I/III collagen matrix	15; 10 M, 5F (48)	24–38	Coverage of chondral defect	[77]
BM-MSC seeded on type I collagen scaffold supplemented with fibrin glue	2; 2 M (24–25)	30–31	Partial coverage of chondral defect	[78]
Peripheral blood-derived MSC with HA	5; 1 M, 4F (39 ± 11)	10–26	Partial coverage of chondral defect	[79]
BMDC within fibrin glue and coverage with collagen and collagen membrane	1; M; 37 yrs	24	Partial coverage of chondral defect	[80]
BMDC in fibrin glue and coverage with a PGA + HA membrane	9; 5 M, 4F (48 ± 9)	20–24	Hyaline-like cartilage	[81]
BMDC in collagen/platelet paste or seeded on HA or seeded on HA scaffold supplemented with platelet gel	49; 27 M, 22F (28 ± 9)	48	Coverage of chondral defect in 45%	[59]
Peripheral blood-derived MSC and HA	49; 17 M, 32F (37 ± 7)	24	Partial coverage of chondral defect	[18]

*BM-MSC* bone marrow-derived mesenchymal stem cells, *PLA* polylactic acid, *HA* hyaluronic acid, *PGA* polyglycolic acid

### Optimization of MSC implantation tool required for the repair of articular cartilage

Our in vitro study showed that MSCs seeded on PLGA plug can differentiate into chondrocytes in the growth medium alone, even when MSCs were not cultured in chondrocyte differentiation medium [40]. In order to avoid improper cell differentiation, e.g., osteoblast cells that can trigger ectopic calcification, a special vehicle is needed in advance to direct MSCs into chondrocyte differentiation.

Various mechanisms have been proposed for the differentiation of MSC into chondrocytes. In vitro studies showed that TNF-α, IL-1β, and IL-17 suppress MSC differentiation into chondrocytes [60–64]. Specifically, TNF-α and IL-1β inhibit the smad signaling pathway, and concomitantly down-regulate *Sox9* gene, which encodes master transcriptional factor required for chondrocyte differentiation [61, 62]. On the other hand, IL-17 inhibits the activity of protein kinase A (PKA), leading to low phosphorylation level of SOX9, which consequently inactivate SOX9 [64]. Taken together, pro-inflammatory cytokines do not only inflict damage of joints, but also suppress MSC differentiation into chondrocytes. Notably, stimulation of MSCs, which produce high levels of IL-6, with IL-6R results in the activation of IL-6/IL-6R signaling, which in turn induces the expression of various cartilage-related genes in MSCs, resulting in MSC differentiation into chondrocytes [65].

Based on the above information, it is interesting to study whether implantation of PLGA and IL-6R-treated MSCs contributes to the repair of articular cartilage.

## Conclusions

There is a disadvantage in using osteochondral repair as the goal of treatment of articular cartilage tissue damage, since such strategy can negatively affect the residual healthy cartilage tissue. New methods of MSC-based therapy have been tried for the repair of articular cartilage damage. In vitro studies demonstrated that MSCs can differentiate into chondrocytes. Further, 3D culture applied with scaffold enhanced differentiation of MSCs into chondrocytes. In animal models of cartilage damage, the use of local implantation system comprising scaffolds with MSCs, such as PLGA and HA, can result in repair of the articular cartilage with the formation of new hyaline cartilage-like tissue. Furthermore, implantation of MSCs seeded on scaffold into the damaged articular cartilage of patients resulted in histopathological improvement with regeneration of the cartilage tissue. Further studies are necessary to find optimal implantation vehicles that can result in regeneration of articular cartilage.

## Abbreviations

ADL: Activity of daily life
AIA: Antigen-induced arthritis
BMDC: Bone marrow-derived cell
HA: Hyaluronic acid
IL-6R: Interleukin-6 receptor
MSCs: Mesenchymal stem cells
PGA: Polyglycolic acid
PLA: Polylactic acid
PLGA: Poly-lactic and co-glycolic acids
QOL: Quality of life
RA: Rheumatoid arthritis
TCP: Tricalcium phosphate
